# Supplementary material for: Developmental Parallels Between the Human Organs of Zuckerkandl and Adrenal Medulla
Source: Life (Basel). 2025 Jul 31;15(8):1214. doi: 10.3390/life15081214 (PMC12387564; doi:10.3390/life15081214)
Supplement: Supplementary file 1 [file life-15-01214-s001.zip › life-3674568-supplementary.pdf]

Supplement

Table S1. Characteristics of material.

Case No.	Gestational Age	Clinical data
1–2 (dichorionic diamniotic twins)	8–9 g.w.[1]	Uterine scar after 2 cesarean sections, retrochorionic hematoma
3*	9 g.w.	Chorion ingrowth into the uterine scar clinically. During the histological investigation of embryonic material, huge microcalcifications were found in the brain, spinal cord, and ribs of unknown nature. Pathologies in other organs were not found.
4	9–10 p.c.w.[2] (11–12 g.w.)	Deficiency of the uterine scar after 3 cesarean sections
5	11–12 g.w. (9–10 p.c.w.)	Deficiency of the uterine scar after 3 cesarean sections, detachment of the ovum
6	12 g.w.	Cervical carcinoma TisNxM0, small uterine leiomyoma, spontaneous abortion
7	16 g.w.	Spontaneous abortion. Anamnesis of cervix amputation 5 years before due to carcinoma of the cervix in situ.
8	20 g.w.	Primary fetoplacental insufficiency, preterm rupture of membranes, anhydramnion, induced abortion, uterine scar from cesarean section 1 year before, miscarriage, gestational diabetes mellitus, multiple uterine leiomyomas.
9	20 g.w.	Multiple leiomyomas of the uterus, late induced abortion
10	20–21 g.w.	Complete hydatidiform mole of 1 of the twins
11	21 g.w.	COVID-19, systemic lupus erythematosus in mother
12*	21–22 g.w.	Termination of pregnancy for fetal reasons: spina bifida, Arnold-Chiari Syndrome, placental haematoma, toxic goiter (medical correction), colpitis, caused by *Trichomonas vaginalis*, uterine scar from 2 cesarean sections 1 and 2 years before.
13	22–23 g.w.	Severe preeclampsia
14*	25–26 g.w.	Induced abortion for fetal reason: agenesis of the corpus callosum, microencephaly, cortical malformation, schizencephaly. Chronic cytomegalovirus infection in the mother. At whole exome sequencing, a heterozygous pathogenic variant of the ARX gene on the X chromosome was found. Pathologies in other organs and systems except for nervous system were not found.

[1] – Gestation weeks. [2] – Postconceptional weeks.

*We believe that the clinical conditions described in these cases likely did not significantly impact the results regarding the development of the AM and OZ, as no pathologies were detected in organs and systems other than the central nervous system and skeletal system in case No. 3. However, we cannot completely rule out the possibility of some influence.

Table S2. Results of immunohistochemical reactions.

Case No.	Gestational Age	TH	DBH	PNMT	βIII-tubulin	S100
1-2 (dichorionic diamniotic twins)	8-9 g.w.	**LCs**[1] ExA[3]+, InA[4]+;<br><br>**SCs**[2] ExA and InA+(↓)/-;	**LCs** ExA+, InA +;<br><br>**SCs** ExA and InA +(↓)/-	-	**LCs** ExA and InA+;<br><br>**SCs** ExA and InA +	**C-N**[6]+(↓)
3	9 g.w.	**LCs** ExA+, InA+;<br><br>**SCs** ExA and InA +(↓)/-;	**LCs** ExA+, InA +;<br><br>**SCs** ExA and InA +(↓)/-	-	**LCs** ExA and InA+;<br><br>**SCs** ExA and InA +	**C-N+**<br>**C-LC**[7]+<br>**C-SC**[8]+
4	9-10 p.c.w. (11-12 g.w.)	**LCs** ExA+, InA+;<br><br>**SCs** ExA and InA +(↓)/-;	**LCs** ExA+, InA +;<br><br>**SCs** ExA and InA +(↓)/-	-	**LCs** ExA and InA +;<br><br>**SCs** ExA and InA+	**C-N+**<br>**C-LC+**<br>**C-SC+**
5	11-12 g.w. (9-10 p.c.w.)	**LCs** ExA+, InA+;<br><br>**SCs** ExA and InA+(↓)/-;	**LCs** ExA+, InA +;<br><br>**SCs** ExA and InA +(↓)/-	-	**LCs** ExA and InA+;<br><br>**SCs** ExA and InA +	**C-N+**<br>**C-LC+**<br>**C-SC+**
6	12 g.w.	**LCs** ExA+, InA+;<br><br>**SCs** ExA +; InA+(↓)	**LCs** ExA+, InA+;<br><br>**SCs** ExA +; InA+(↓)	**LCs** InA +	**LCs** ExA and InA +;<br><br>**SCs** ExA and InA +	**C-N+**<br>**C-LC+**<br>**C-SC+**
7	16 g.w.	**LCs** ExA+, InA+;<br><br>**SCs** InA+(↓)	**LCs** ExA+, InA+;<br><br>**SCs** InA+(↓)	-	**LCs** ExA and InA +;<br><br>**SCs** ExA and InA +	**C-N+**<br>**C-LC+**<br>**C-SC+**
8	20 g.w.	**LCs** ExA+, InA+;<br><br>**SCs** InA+(↓);<br><br>**N(G)**[5]+	**LCs** ExA+, InA+;<br><br>**SCs** InA+(↓);<br><br>**N(G)+**	-	**LCs** ExA +; InA-/+(↓);<br><br>**SCs** ExA and InA +	**C-N+**<br>**C-LC+**<br>**C-SC** InA+
9	20 g.w.	**LCs** ExA+, InA+;<br><br>**SCs** InA+(↓);<br><br>**N(G)**[5]+	**LCs** ExA+, InA+;<br><br>**SCs** InA+(↓);<br><br>**N(G)+**	**LCs** InA -/↓	**LCs** ExA +; InA -/+(↓);<br><br>**SCs** ExA and InA +	**C-N+**<br>**C-LC+**<br>**C-SC** InA+

10	20-21 g.w.	**LCs** ExA**+**, InA**+**;<br>**SCs** InA+**(↓)**;<br>**N(G)+**	**LCs** ExA**+**, InA+;<br>**SCs** InA+**(↓)**;<br>**N(G)+**	**LCs** InA +	**LCs** ExA **+(↓);** InA **-/+(↓)**;<br>**SCs** ExA and InA **+**	**C-N+**<br>**C-LC+**<br>**C-SC** InA+
11	21 g.w.	**LCs** ExA+, InA+;<br>**SCs** InA+**(↓)**;<br>**N(G)+**	**LCs** ExA**+**, InA+;<br>**SCs** InA+**(↓)**;<br>**N(G)+**	**LCs** InA +	**LCs** ExA **+;** InA -/+**(↓)**;<br>**SCs** ExA and InA **+**	**C-N+**<br>**C-LC+**<br>**C-SC** InA+
12	21-22 g.w.	**LCs** ExA+, InA+;<br>**SCs** InA+**(↓)**;<br>**N(G)+**	**LCs** ExA+, InA+;<br>**SCs** InA+**(↓)**;<br>**N(G)+**	**LCs** InA +	**LCs** ExA+; InA+**(↓)**/-;<br>**SCs** ExA and InA **+**	**C-N+**<br>**C-LC+**<br>**C-SC** InA+
13	22-23 g.w.	**LCs** ExA+, InA+;<br>**SCs** InA+**(↓)**;<br>**N(G)+**	**LCs** ExA+, InA+;<br>**SCs** InA+**(↓)**;<br>**N(G)+**	**LCs** InA +	**LCs** ExA **+(↓)** InA **-**;<br>**SCs** InA +	**C-N+**<br>**C-LC+**<br>**S-SC** InA+
14	25-26 g.w.	**LCs** ExA+, InA+;<br>**SCs** InA+**(↓)**;<br>**N(G)+**	**LCs** ExA+, InA+;<br>**SCs** InA+**(↓)**;<br>**N(G)+**	**LCs** InA +	**LCs** ExA+**(↓)** InA **-/+(↓)**;<br>**SCs** InA +	**C-N+**<br>**C-LC+**<br>**C-SC** InA+

[1] - large cells (**LCs**). [2] – small cells (**SCs**). [3] – extraadrenally. [4] – intraadrenally. [5] – neurons in ganglia (**N(G)**), [6] – cells associated with nerves (**C-N**), [7] – cells at periphery/among LCs (**C-LC**), [8] - cells at periphery/among SCs (**C-SC**). "**+**" – strong positive reaction with antibody, "**-**"– negative reaction with antibody, ↓ - weak positive reaction with antibody.

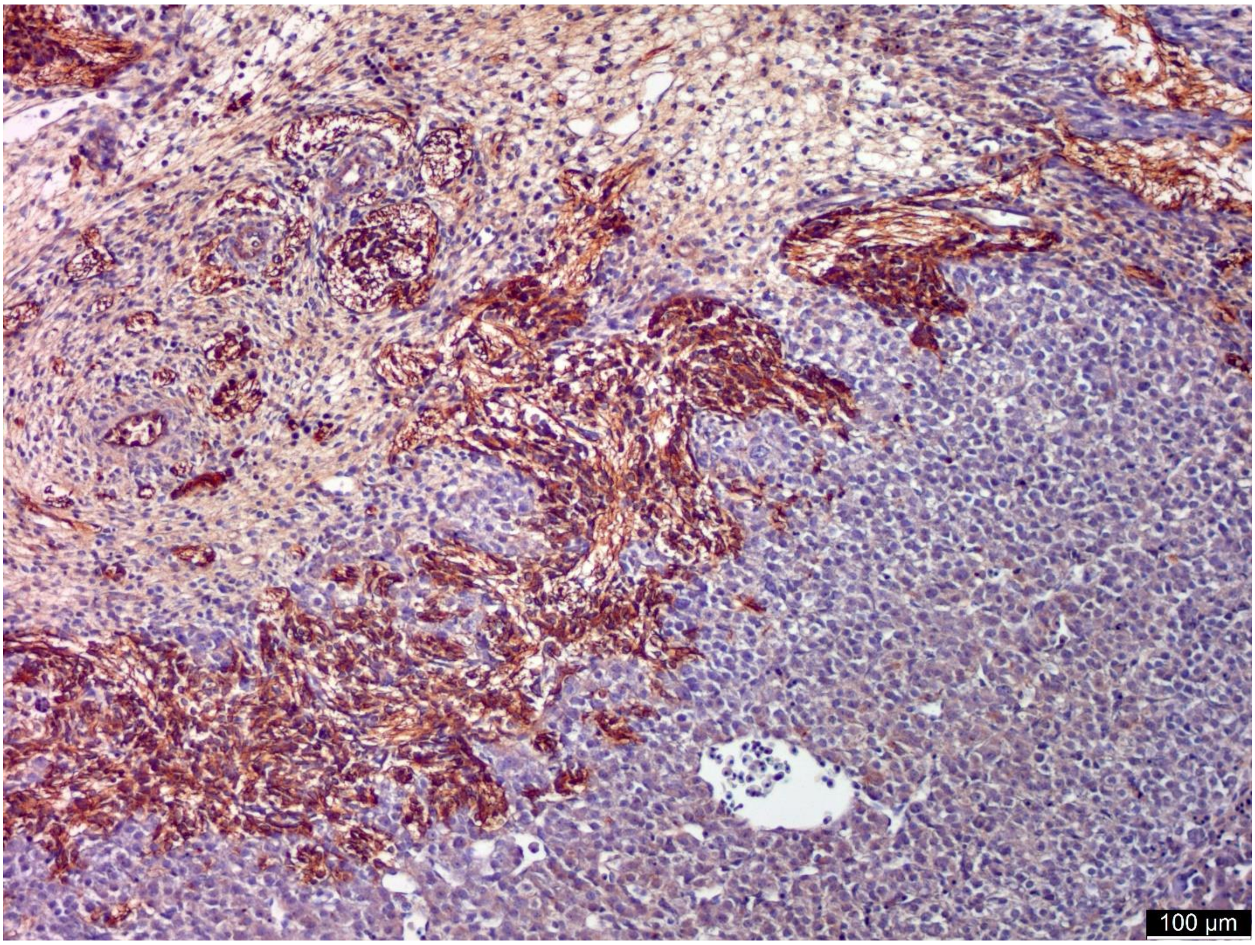

Figure S1. 8-9 g.w. (No 1). The adrenal anlage with surrounding tissues. IHC for βIII-tubulin.

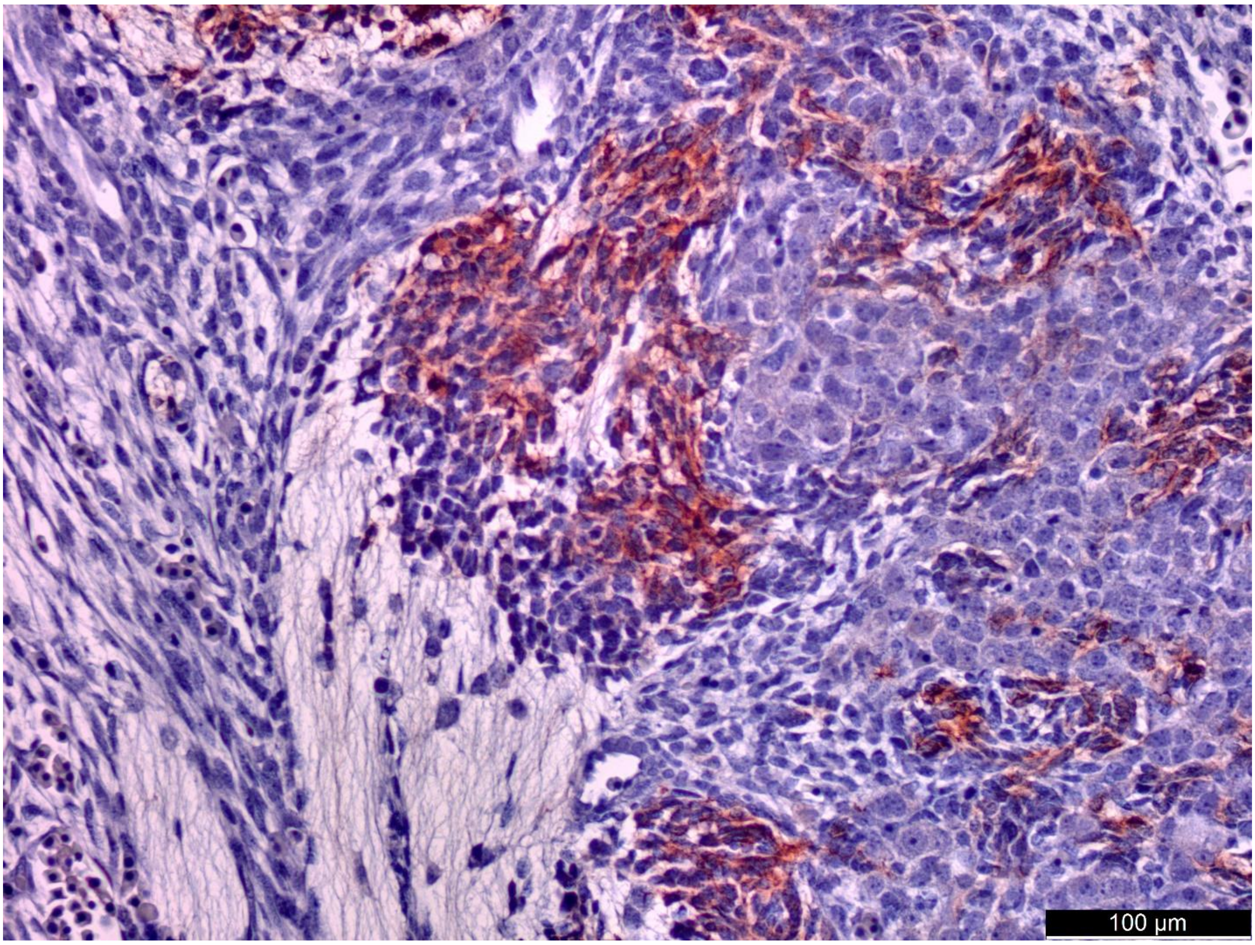

Figure S2. 8-9 g.w. (No 2). The adrenal anlage with the adjacent nerve. IHC for DBH.
